# Predictive value of peripheral blood immune markers for castration-resistant prostate cancer development after endocrine therapy

**DOI:** 10.3389/fonc.2026.1696687

**Published:** 2026-02-02

**Authors:** Fengshan Li, Luwei Cui, Guanlan Zhang, Jianwei Hao

**Affiliations:** 1 Department of Medicine, Graduate School, Henan University, Kaifeng, China; 2 Department of Urology, Henan Provincial People’s Hospital, Zhengzhou, China; 3 Department of Urology and Wuxi School of Medicine, Jiangnan University Medical Center, Wuxi, Jiangsu, China

**Keywords:** castration-resistant prostate cancer, endocrine therapy, nomogram prediction model, PLR, SII, survival analysis

## Abstract

**Objective:**

To evaluate the predictive value of peripheral blood immune markers for progression to castration-resistant prostate cancer (CRPC) in prostate cancer (PCa) patients undergoing endocrine therapy.

**Methods:**

This retrospective study included 106 PCa patients treated with endocrine therapy between 2021 and 2024. Peripheral blood immune parameters were compared between patients who did and did not experience progression to CRPC (progression and stable groups, respectively). The identified independent predictors of CRPC were then used to construct a nomogram for the identification of high-risk patients for early intervention. Internal validation was performed using the bootstrap method with 1000 resamples. Nomogram performance was evaluated by receiver operating characteristic curve, calibration curve, and decision curve analyses.

**Results:**

Univariate analysis showed significant differences (*P* < 0.05) in prostate-specific antigen level, Gleason score, neutrophil-to-lymphocyte ratio, platelet-to-lymphocyte ratio (PLR), monocyte-to-lymphocyte ratio, systemic immune inflammation index (SII), and pan-immune inflammation value (PIV), CD4+/CD8+ T cell ratio, and T4 stage between the progression and stable groups. Multivariate analysis further identified the PLR, SII, CD4+/CD8+ T cell ratio, and T4 stage as independent risk factors for CRPC development. The developed predictive model demonstrated strong predictive performance (area under the curve =0.934, Hosmer–Lemeshow *P*>0.05). The c-index for internal validation was 0.914, further confirming the predictive performance of the model. Employing clinically optimized thresholds, survival analysis confirmed that a PLR ≥140, SII ≥520, and CD4+/CD8+ T cell ratio <1.6 could significantly predict the 2-year CRPC risk.

**Conclusion:**

The PLR, SII, CD4+/CD8+ T cell ratio, and T4 tumor stage are independent risk factors for CRPC progression within 2 years of endocrine therapy. The survival prediction model based on these factors offers good predictive efficacy.

## Introduction

Prostate cancer currently ranks as the second most frequently diagnosed cancer and the fifth leading cause of cancer-related death among men worldwide, with a global incidence rate that continues to steadily increase (1). Androgen deprivation therapy (ADT) is the standard treatment for metastatic prostate cancer and is achieved through surgical castration or pharmacological inhibition of the androgen pathway (2). Clinical data indicate that while prostate-specific antigen levels can be effectively controlled initially in most patients with prostate cancer, approximately half of cases will develop castration resistance within 2–3 years of disease progression, ultimately progressing to metastatic castration-resistant prostate cancer (CRPC) (3). The development of metastatic CRPC signifies the progression of the disease to its terminal stage, which presents a major challenge in current clinical management. Numerous studies have found that earlier progression of prostate cancer to the CRPC stage corresponds to a poorer prognosis and shorter survival duration (4), thus delaying the progression of patients to CRPC will help prolong the overall survival time of prostate cancer patients (5, 6). Tumor development and progression involve multidimensional biological regulatory mechanisms. The complexity of these processes suggests that a wide array of potential predictive indicators exists but also that any single factor is unlikely to provide an effective prognostic biomarker. Recent studies have confirmed that the host immune defense system plays a pivotal role in the pathological evolution of malignant tumors. Interdisciplinary studies integrating tumor etiology, molecular cell biology, and immunology have revealed that malignant proliferating cells, immune effector cells, and their secreted cytokine networks collectively constitute the tumor immune microenvironment. This dynamic system forms a sophisticated molecular network that regulates host immune responses through intercellular signaling and interaction with microenvironmental factors. Lymphocytes play an important role in antitumor immunity by triggering apoptosis among tumor cells and inhibiting their proliferation and migration in various stages of cancer progression (7). Moreover, studies have shown that a variety of inflammatory markers can serve as potential prognostic biomarkers for various malignancies (8–12), and previous studies have identified the predictive potential of serum immune-inflammatory cells for tumor prognosis and response to treatment (13). The immune–inflammatory index also represents a minimally invasive and readily accessible biomarker with prognostic value across a wide variety of cancers (14). With recent breakthroughs in technologies such as single-cell sequencing and liquid biopsy, research on peripheral blood immune biomarkers has entered a new phase, showing substantial potential for early cancer screening, prediction of treatment response, and prognostic assessment.

In recent years, substantial progress has been achieved in the precision management of CRPC. The 2025 NCCN and EAU guidelines identify molecular biomarkers derived from tissue or liquid biopsies—such as androgen receptor splice variant 7 (AR-V7) and BRCA/homologous recombination repair (HRR) gene mutations—as well as advanced imaging modalities as core tools for risk stratification and treatment decision-making in CRPC, with demonstrated value for predicting treatment response, guiding targeted therapies (e.g., PARP inhibitors), and assessing prognosis (15–20). However, these molecular assays and advanced imaging approaches often require specialized technological platforms, incur high costs, and are not well suited for frequent or dynamic monitoring in routine clinical practice. In contrast, peripheral blood immune–inflammatory indices, such as the neutrophil-to-lymphocyte ratio (NLR), platelet-to-lymphocyte ratio (PLR), and systemic immune-inflammation index (SII), derived from routine blood tests and lymphocyte subset analyses offer clear advantages in terms of cost-effectiveness, accessibility, and reproducibility, enabling non-invasive and dynamic assessment of systemic inflammatory status and immune balance (21–25). Therefore, exploring these readily available immune indicators as complementary tools to guideline-recommended molecular biomarkers may have substantial clinical and practical value for early risk stratification and dynamic, comprehensive management of CRPC across diverse clinical settings, particularly in resource-limited contexts or when longitudinal monitoring is required. Therefore, the present study aimed to investigate the predictive value of peripheral blood immune biomarkers for progression to CRPC in patients with prostate cancer undergoing endocrine therapy. The results suggest that such biomarkers hold significant promise for risk stratification, therapeutic decision-making, and prognosis improvement for these patients.

## Patients and methods

### Patients

This retrospective study analyzed data from 106 patients hospitalized at Henan Provincial People’s Hospital between January 2021 and December 2024 who were pathologically diagnosed with prostate cancer and underwent endocrine therapy. All patients received endocrine therapy according to the following protocol: chemical castration (goserelin 3.6 mg or leuprolide 3.75 mg administered subcutaneously once monthly; or triptorelin 3.75 mg administered intramuscularly once monthly; or goserelin 10.8 mg administered subcutaneously every 3 months) combined with oral enzalutamide 160 mg once daily. The exclusion criteria were as follows (1): failure to provide informed consent (2); failure to take medication as scheduled or discontinuation of medication without authorization during treatment (3); incomplete clinical data, poor compliance, or incomplete follow-up data (4); any blood disorder, immune system disease, infectious disease, cardiovascular disease, or other condition that may affect the results of immune inflammatory marker tests (5); concurrent urinary tract or respiratory tract infection, any other systemic infectious disease, or any acute or chronic infectious disease (6); recent history of blood transfusion, blood donation, radiation therapy, or immunotherapy; and (7) a prior history of other malignancies. This study was approved by the Ethics Committee of Henan Provincial People’s Hospital and was performed in compliance with the Declaration of Helsinki. Written informed consent was waived due to the retrospective nature of the study.

### Data collection and grouping

This study retrospectively reviewed the clinical data and test results for hematological parameters of patients at the time of initial treatment, including (1): demographic and oncological characteristics (age, body mass index [BMI], smoking and drinking history, history of underlying diseases, TNM staging, Gleason score, and prostate-specific antigen [PSA] level); and (2) hematological parameters (white blood cell count, monocyte count, neutrophil count, lymphocyte count, platelet count, and levels of immune lymphocyte subsets) and six calculated immune–inflammatory indices based on hematological parameters (NLR, PLR, neutrophil-to-monocyte ratio [NMR], monocyte-to-lymphocyte ratio [MLR], SII, and pan-immune inflammation value [PIV].

Patients who experienced progression to CRPC during endocrine therapy were assigned to the progression group, and all others were included in the stable group. CRPC diagnoses were made in strict adherence to the standardized criteria of the Chinese Guidelines for Prostate Cancer Diagnosis and Treatment (1): serum androgen levels meeting the castration threshold (testosterone concentration <1.7 nmol/L); and (2) objective evidence of disease progression based on either biochemical progression manifested as a stepwise increase in PSA levels over three consecutive tests with an interval of ≥1 week, with the last test exceeding 2 µg/L and representing an increase by >50% from the baseline minimum value or imaging progression defined as bone scans showing at least two new metastatic lesions or enlargement of soft tissue lesions meeting the criteria for solid tumor response evaluation (26).

### Development and evaluation of predictive nomogram

To construct a nomogram capable of accurately predicting the risk of CRPC progression in patients with prostate cancer, we employed univariate analysis followed by multivariate logistic regression analysis to identify factors associated with the occurrence of CRPC. In the multivariate analysis, forward selection was used to screen for independent predictors, and the significant variables were included in the construction of the risk chart. The discriminatory ability of the model was assessed by plotting the receiver operating characteristic (ROC) curve and calculating the area under the curve (AUC). Calibration curves were used to evaluate the consistency between the predicted and actual observed probabilities of CRPC risk. Decision curve analysis (DCA) was performed to quantify the clinical net benefit of applying this nomogram in practice. Model validation was conducted using the bootstrap method for internal validation, with B = 1000 repetitions.

### Statistical analysis

All analyses were performed using SPSS 27.0 and RStudio 4.2.2. The normality of continuous variables was assessed using the Shapiro–Wilk test. Normally distributed data are presented as mean ± standard deviation (SD) and were compared between groups using the independent-samples t-test, whereas non-normally distributed data are expressed as median (interquartile range). Categorical variables are expressed as frequencies (percentages), and comparisons between groups were performed using the chi-square test or Fisher’s exact test. Variables found to be significant on the univariate analyses were entered into a multivariate logistic regression model, and stepwise selection was used to identify independent predictors of progression to CRPC. Including the identified factors, a nomogram was constructed with the rms package. Its discriminative ability was quantified by calculation of Harrell’s C-index, and calibration curves were plotted to assess the goodness-of-fit of the model. Bootstrap resampling with 1,000 replicates was employed for internal validation to correct for overoptimism in model performance estimates. Optimal cut-off values were determined using X-tile, and ROC curves were used to evaluate predictive accuracy. Kaplan–Meier curve analysis with log-rank tests was performed to compare CRPC progression-free survival (PFS) among subgroups. Hazard ratios (HRs) with 95% confidence intervals (CIs) were calculated using Cox proportional hazards models to quantify the magnitude of survival differences. A two-sided P<0.05 indicated statistical significance.

## Results

### Clinical characteristics of patients with and without CRPC progression

**Figure 1:**
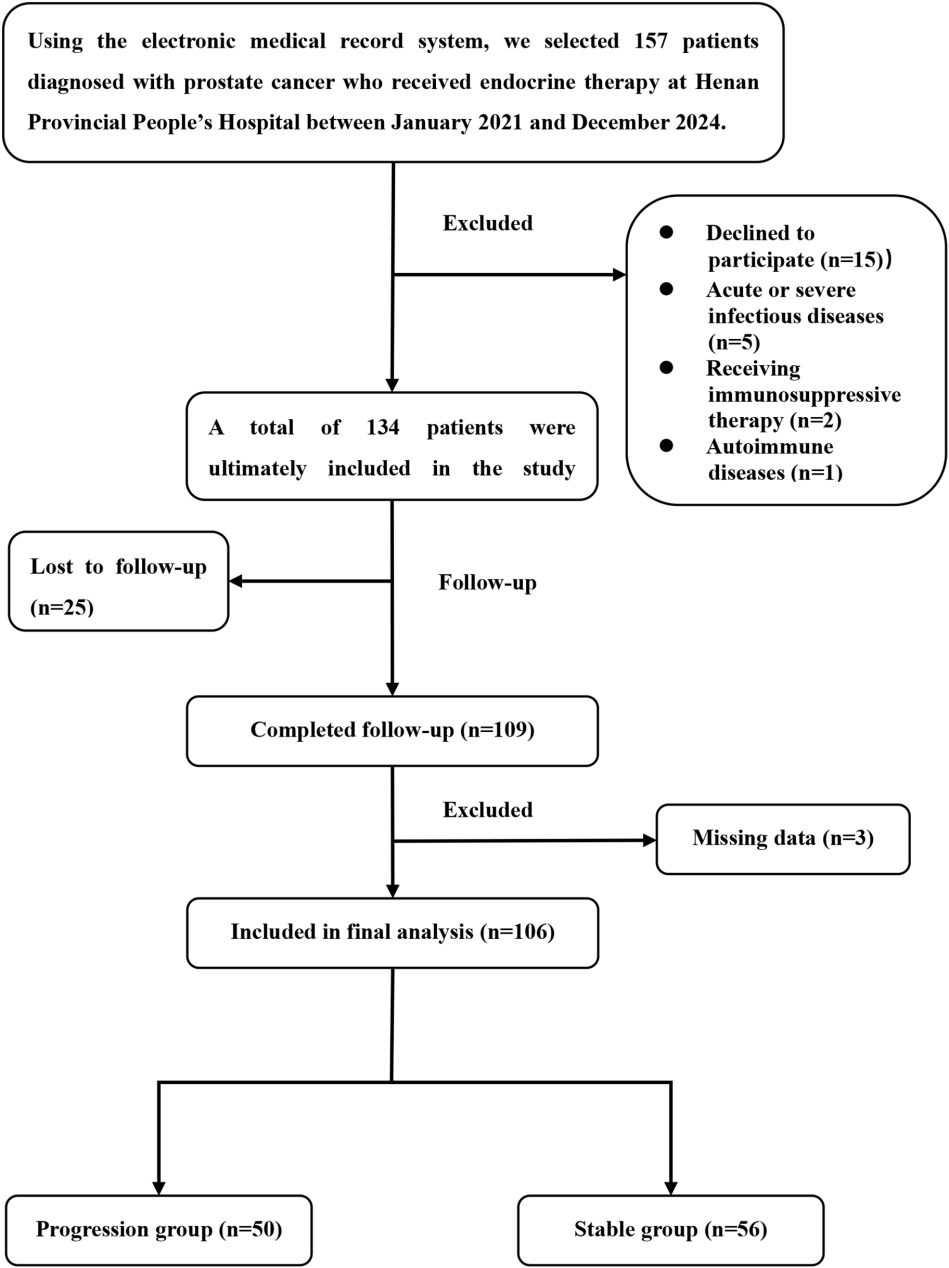
Flow chart of patient selection.

We initially screened 156 cases of prostate cancer treated with endocrine therapy during the study period. After application of the exclusion criteria, the final study population included 106 patients, as detailed in the flow chart in **Figure 1**.

Among the 106 patients included in this study, 50 patients (47.20%) experienced progression to CRPC within 2 years of endocrine and were included in the progression group, while the remaining 56 patients (52.84%) did not and were included in the stable group. The clinical characteristics and immune–inflammatory parameters of the total study population are presented in **Tables 1** and **2**, respectively.

**Table 1:** Comparison of clinical characteristics between the progression and stable groups.

Indicator	Whole cohort (n=106)	Progression group (n=50)	Stable group (n=56)	P
Age (years)	71.00 (67.00, 77.00)	71 (67, 79)	71 (67, 76)	0.628
BMI (kg/m^2^)	25.25 (23.28, 26.85)	24.91 (23.02, 27.04)	25.59 (23.59, 26.78)	0.423
Smoking history, n (%)				0.729
Yes	49 (46.23%)	24 (48.00%)	25 (44.64%)	
No	57 (53.77%)	26 (52.00%)	31 (55.36%)	
Drinking history, n (%)				0.290
Yes	46 (43.40%)	19 (38.00%)	27 (48.21%)	
No	60 (56.60%)	31 (62.00%)	29 (51.79%)	
Hypertension, n (%)				0.747
Yes	42 (39.62%)	19 (38.00%)	23 (41.07%)	
No	64 (60.38%)	31 (62.00%)	33 (58.93%)	
Diabetes mellitus				0.943
Yes	23 (21.70%)	11 (78.00%)	12 (21.43%)	
No	83 (78.30%)	39 (22.00%)	44 (78.57%)	
Gleason score, n (%)				<0.001
≤8	49 (46.23%)	14 (28.00%)	35 (62.50%)	
>8	57 (53.77%)	36 (72.00%)	21 (37.50%)	
T stage, n (%)				0.002
T2	35 (33.02%)	10 (20.00%)	25 (44.64%)	
T3	37 (34.91%)	16 (32.00%)	21 (37.50%)	
T4	34 (32.07%)	24 (48.00%)	10 (17.86%)	
N stage, n (%)				0.096
N0	64 (60.38%)	26 (52.00%)	38 (67.86%)	
N1	42 (39.62%)	24 (48.00%)	18 (32.14%)	
M stage, n (%)				0.226
M0	20 (18.87%)	7 (14.00%)	13 (23.21%)	
M1	86 (81.13%)	43 (86.00%)	43 (76.79%)	
PSA, n (%), ng/mL				0.020
<20	22 (20.75%)	11 (22.00%)	11 (20.00%)	
20-100	28 (26.42%)	7 (14.00%)	21 (38.00%)	
>100	56 (52.83%)	32 (64.00%)	24 (43.00%)	

**Table 2:** Comparison of immune–inflammatory indicators between the progression and stable groups.

Indicator	Whole cohort (n=106)	Progression group (n=50)	Stable group (n=56)	P
White blood cell count (×10^9^/L)	6.26 (4.96, 7.20)	5.97 (4.97, 7.07)	6.42 (5.01, 7.22)	0.365
Neutrophil count (×10^9^/L)	4.12 (3.37, 4.90)	4.24 (3.55, 5.26)	3.56 (3.07, 4.36)	0.009
Lymphocyte count (×10^9^/L)	1.71 (1.33, 2.09)	1.44 (1.20, 1.72)	1.95 (1.59, 2.20)	<0.001
Monocyte count (×10^9^/L)	0.43 (0.32, 0.51)	0.45 (0.31, 0.56)	0.41 (0.35, 0.47)	0.175
Platelet count (×10^9^/L)	198.00 (168.00, 230.00)	205.50 (184.50, 239.25)	187.50 (158.50, 226.25)	0.008
NLR	2.43 (1.97, 3.20)	3.15 (2.41, 3.62)	2.10 (1.58, 2.52)	<0.001
PLR	121.29 (97.95, 142.86)	147.08 (121.62, 173.53)	103.83 (77.19, 122.93)	<0.001
MLR	0.25 (0.18, 0.32)	0.30 (0.25, 0.46)	0.22 (0.15, 0.27)	<0.001
NMR	9.82 (7.00, 12.58)	10.14 (7.45, 13.08)	9.60 (6.98, 11.58)	0.529
SII	486.96 (364.11, 664.89)	618.61 (510.92, 739.87)	377.03 (270.67, 503.14)	<0.001
PIV	203.92 (134.71, 273.37)	253.01 (175.86, 407.26)	161.58 (104.18, 214.79)	<0.001
CD3+ T cells (%)	66.91 (58.19, 71.53)	67.34 (61.70, 71.53)	66.32 (57.60, 71.78)	0.517
CD4+ T cells (%)	41.26 (33.40, 47.89)	38.09 (31.60, 46.46)	43.29 (36.49, 49.18)	0.009
CD8+ T cells (%)	23.73 (18.05, 29.12)	28.48 (22.12, 36.25)	21.74 (17.63, 25.01)	<0.001
CD4+T/CD8+ T cell ratio	1.63 (1.31, 2.24)	1.50 (0.98, 2.04)	1.90 (1.38, 2.33)	<0.001
CD19+ B cells (%)	11.33 (6.88, 14.58)	10.60 (6.41, 13.81)	11.50 (8.14, 15.09)	0.615
Natural killer cells (%)	19.69 (12.01, 25.54)	17.50 (11.10, 24.08)	20.98 (14.94, 28.12)	0.141

The clinical characteristics of the patients in the two groups are compared in **Table 1**. No significant differences in age, BMI, smoking history, drinking history, diabetes, or hypertension were observed between the groups (all P>0.05). However, the percentage of patients at T stage, the Gleason score, and the PSA level were significantly higher in the progression group than in the stable group (all P<0.05). On comparison of the immune–inflammatory indices between the groups (**Table 2**), the progression group showed significantly higher values for the neutrophil count, platelet count, NLR, PLR, MLR, SII, and PIV than the stable group (all P<0.05), and a significantly lower lymphocyte count than the stable group (P<0.05). Additionally, the CD4^+^ T-cell proportion as well as the CD4^+^ T/CD8^+^ T ratio in the progression group was significantly lower than that in the stable group (P<0.05), whereas the CD8^+^ T-cell proportion was significantly higher than that in the stable group (P<0.05).

### Identification of independent risk factors for progression to CRPC

Variables that differed significantly between the progression and stable groups on the univariate analysis were entered into a multivariate stepwise logistic regression model, using the variable labeling detailed in **Table 3**. The results demonstrated that PLR, SII, CD4^+^/CD8^+^ T cell ratio, and T4 stage were independent risk factors for progression to CRPC within 2 years of endocrine therapy (P<0.05; **Table 4**).

**Table 3:** Variable assignment reference table.

Variable	Factor	Variable labeling
Y	Clinical response	Progression = 1, Stable = 0
X1	T3	T2 = 100
X2	T3	T3 = 010
X3	T4	T4 = 001
X4	PSA	<20 ng/mL= 0; 20–100 ng/mL = 1; 100 ng/mL= 2
X5	Gleason score	≤8分 = 0; >8分 = 1

**Table 4:** Multivariate logistic regression analysis of factors predictive of progression to CRPC.

Factor		Standard error	Wald	P	Odds ratio	95% CI
PLR	0.041	0.016	6.517	0.011	1.042	1.010~1.075
SII	0.005	0.002	4.980	0.026	1.005	1.001~1.009
CD4+T/CD8+ T cell ratio	-1.552	0.521	8.884	0.003	0.212	0.076~0.588
T4 stage	1.777	0.705	6.349	0.012	5.912	1.484~23.548
Constant	-5.279	22.033	6.743	0	0	

### Development and evaluation of the prediction model for progression to CRPC

**Figure 2:**
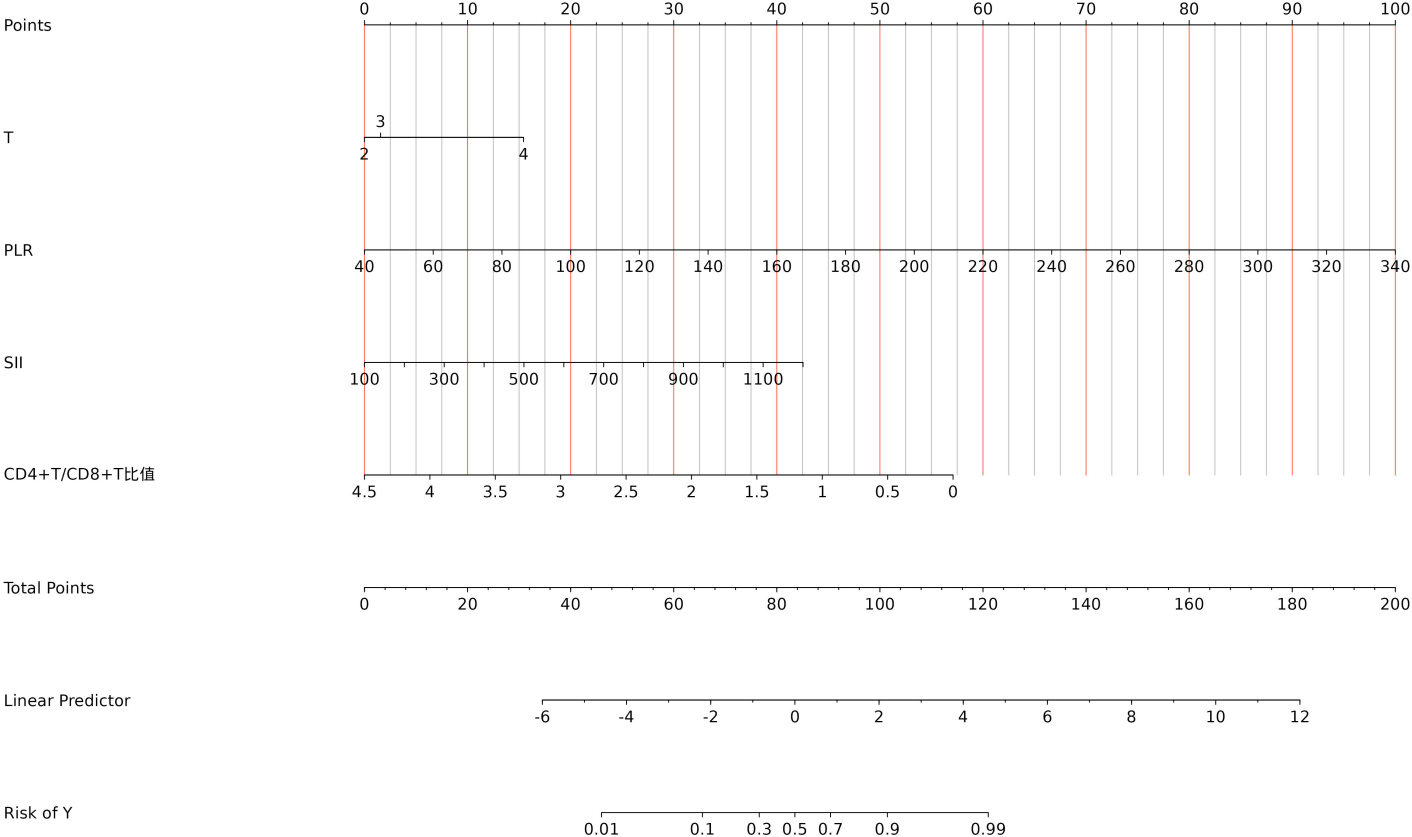
Nomogram for predicting progression to CRPC within 2 years of endocrine therapy in prostate cancer patients.

**Figure 3:**
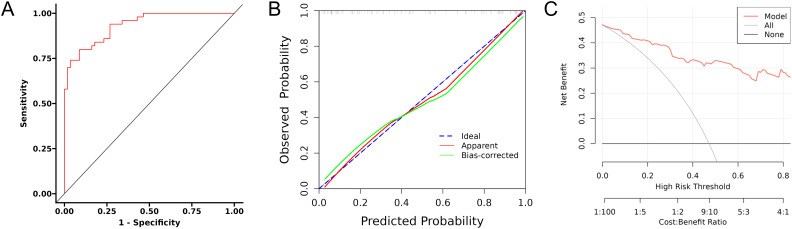
Evaluation of predictive model performance by **(A)** ROC curve analysis, **(B)** calibration curve analysis, and **(C)** DCA.

Using the independent predictors of CRPC identified by the multivariate regression analysis, a predictive model was developed and presented as a visual nomogram (**Figure 2**). The ROC curve for the ability of the model to predict progression of prostate cancer to CRPC within 2 years of endocrine therapy was plotted using R language, as shown in **Figure 3A**. The AUC value for the model was 0.934, indicating good diagnostic performance by the model.

The Hosmer–Lemeshow test was applied to generate a calibration curve for the model (**Figure 3B**). The close proximity of the bias-corrected and apparent lines to the ideal line indicates good consistency, demonstrating that the predictive model has accurate predictive performance. The clinical utility of the model was evaluated by DCA, which quantifies the net benefit of the model to assess its clinical value in real-world medical scenarios. As shown in **Figure 3C**, in the threshold probability range of 0.1–0.7, the model curve significantly surpassed the “None” and “All” reference lines, indicating that the model has good clinical application value within this threshold range. The corrected C‐statistic of the nomogram model obtained from bootstrap resampling was 0.914, indicating good internal validation. Together, these results suggest that the developed nomogram prediction model for CRPC occurrence within 2 years of endocrine therapy in prostate cancer patients has favorable clinical applicability.

### Cutoff values for predictive factors and their association with patient survival

**Figure 4:**
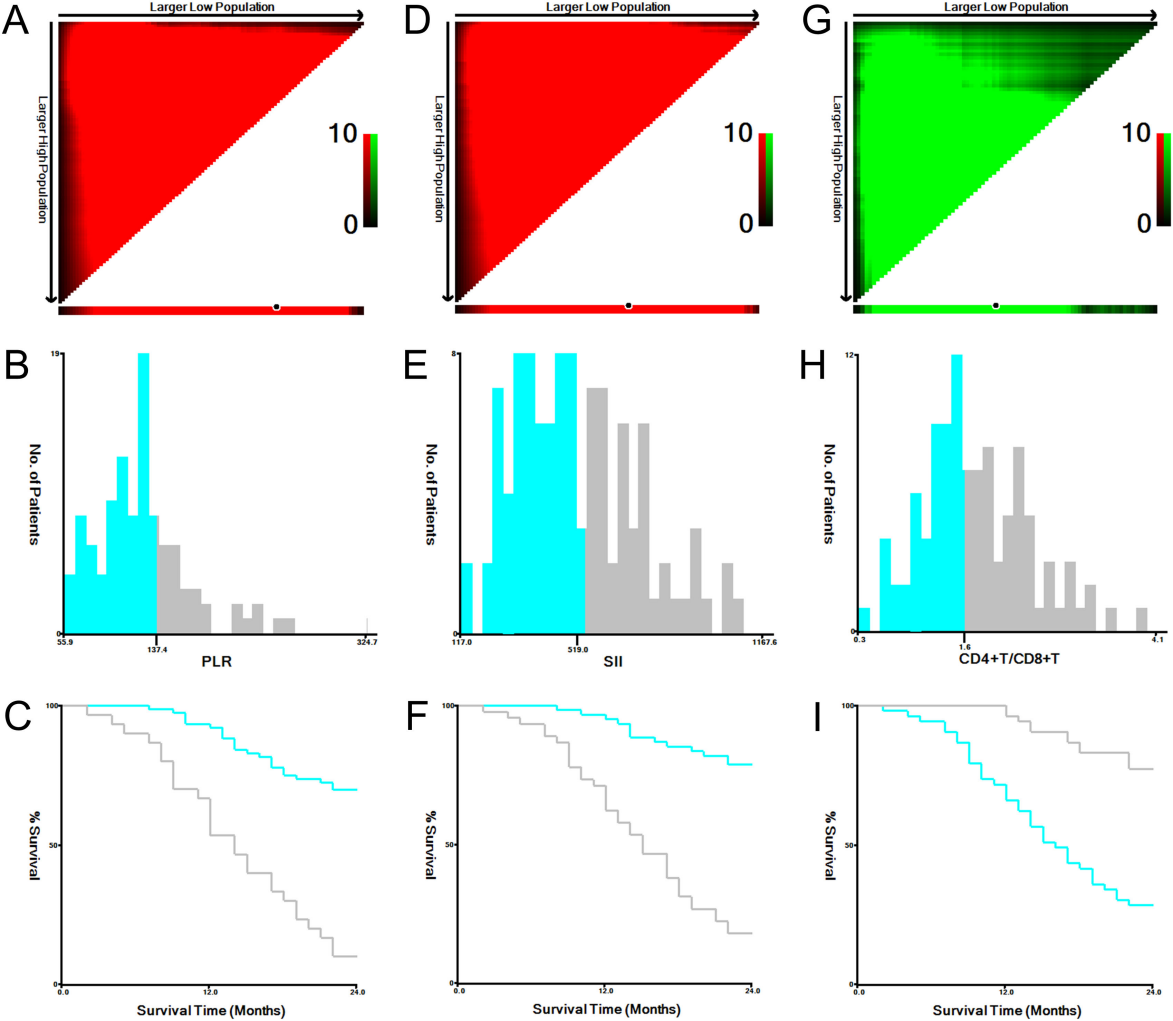
X-tile analysis results for risk factor stratification: two-dimensional survival-risk heatmap for PLR **(A)**, SII **(D)**, and CD4+/CD8+ T cell ratio **(G)**; frequency distribution histogram for PLR **(B)**, SII **(E)**, and CD4+/CD8+ T cell ratio **(H)**; and survival curves after stratification for PLR **(C)**, SII **(F)**, and CD4+/CD8+ T cell ratio **(I)**.

**Figure 5:**
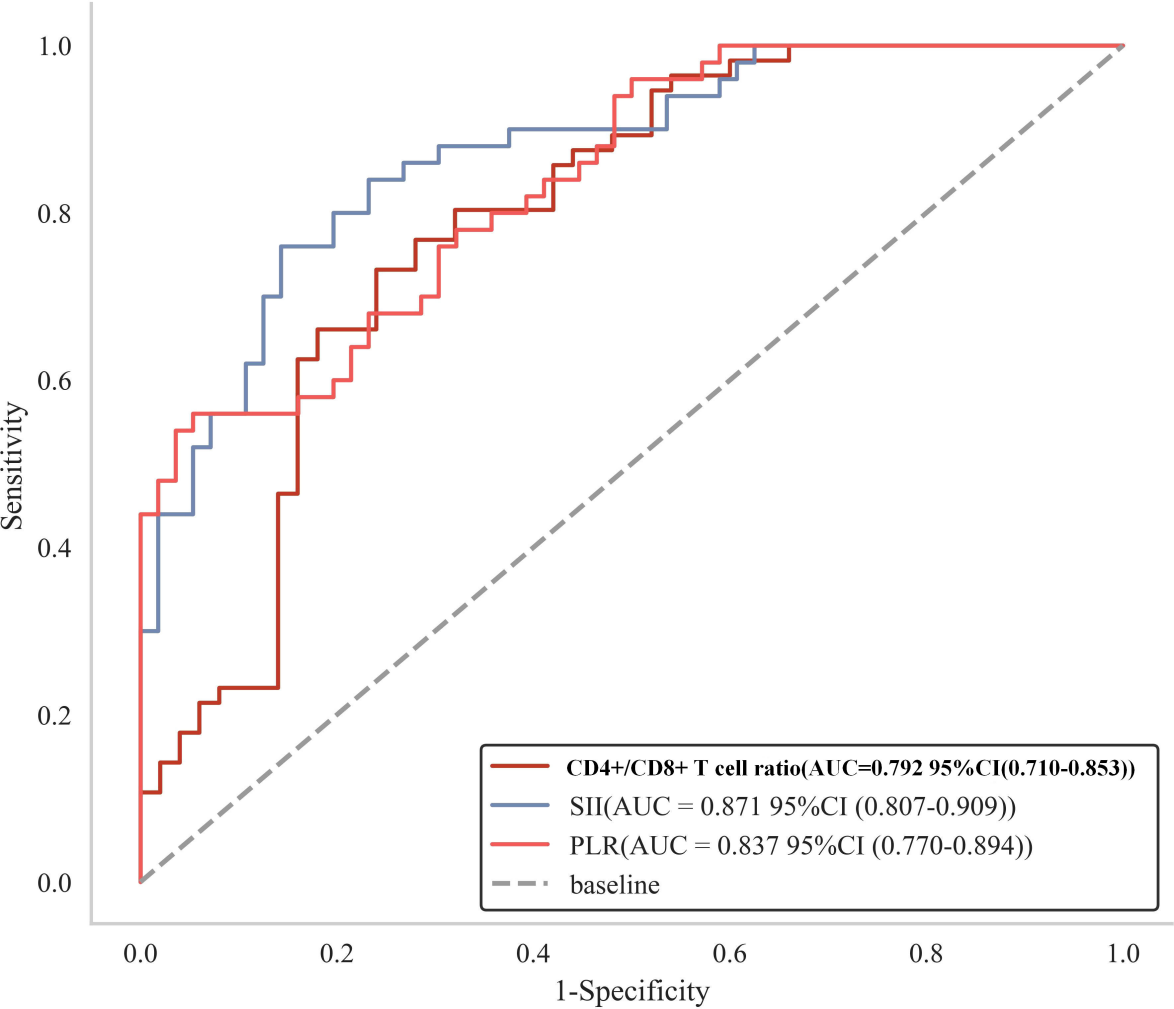
ROC curves for the ability of the calculated cutoff values for PLR, SII, and CD4+/CD8+ T cell ratio to predict CRPC occurrence within 2 years after endocrine therapy in prostate cancer patients.

Using X-tile software, we performed a stratified analysis of three variable factors closely associated with CRPC development and determined the optimal cutoff values for PLR, SII, and CD4+/CD8+ T cell ratio to be 137.405, 518.968, and 1.634, respectively. The predictive efficacy of these indicators for CRPC occurrence in patients with prostate cancer after endocrine therapy was evaluated by constructing ROC curves (**Figures 4**, **5**). To enhance the clinical applicability and reproducibility of the model, these statistically derived cutoffs were optimized to the following practical integer-based thresholds for all subsequent survival analyses: PLR ≥140, SII ≥520, and CD4+/CD8+ T cell ratio <1.6.

**Figure 6:**
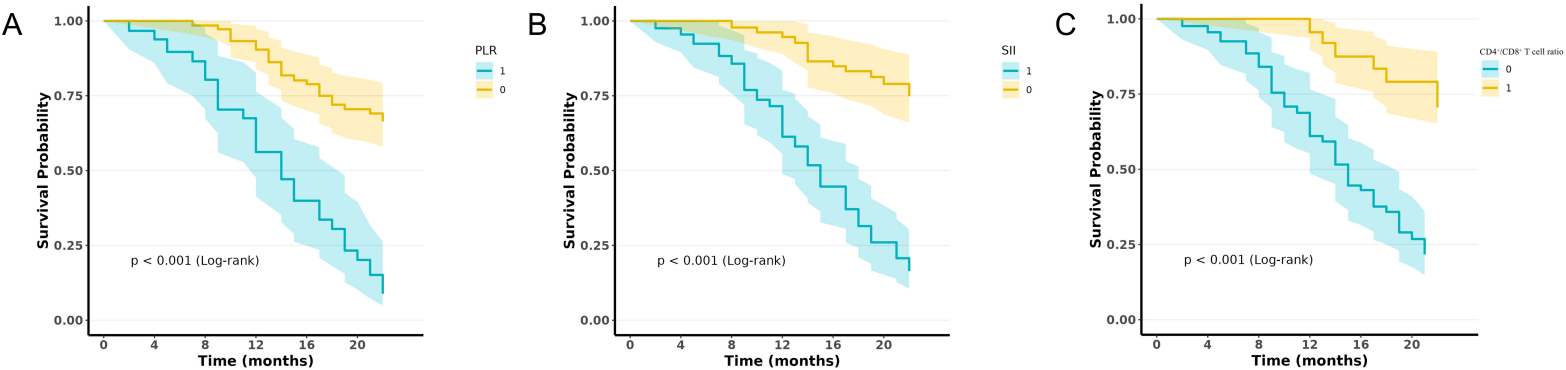
PFS curves for prostate cancer patients in subgroups according to the calculated cutoff values for **(A)** PLR, **(B)** SII, and **(C)** CD4+/CD8+ T cell ratio (log-rank P<0.001).

Based on the clinically optimized thresholds (PLR ≥140, SII ≥520, CD4+/CD8+ T cell ratio <1.6), patients were stratified into high- and low-risk groups. Kaplan–Meier analysis revealed that patients with a PLR ≥140 had significantly shorter progression-free survival (PFS) than those with a PLR <140 (log-rank P<0.001; HR = 2.71, 95% CI: 1.44–5.09; **Figure 6A**). Similarly, patients with SII ≥520 exhibited markedly shorter PFS than those with SII <520 (log-rank P <0.001; HR = 3.29, 95% CI: 1.58–6.85; **Figure 6B**). Conversely, patients with a CD4+/CD8+ T cell ratio ≥1.6 had significantly longer PFS than those with a ratio <1.6 (log-rank P<0.001; HR = 0.32, 95% CI: 0.17–0.63; **Figure 6C**).

## Discussion

Based on the continuous increases in the incidence and mortality of prostate cancer in China (1) and the development of CRPC within 2–3 years of ADT among most patients, which results in a poor prognosis (27), predictive biomarkers for progression to CRPC are urgently needed to support early intervention.

In the present study, multivariate logistic stepwise regression analysis revealed that tumor T-stage served as an independent predictor of CRPC progression within 2 years of endocrine therapy in prostate cancer patients (T4 stage: P = 0.009, OR = 7.430, 95% CI: 1.665–33.148). In contrast, neither the Gleason score nor the baseline PSA level demonstrated a significant correlation with CRPC progression (both P>0.05). Relevant studies have demonstrated that the T stage of prostate cancer is closely associated with disease progression and prognosis, showing that with advancing T stage, patients have an increased risk of distant metastasis, reduced survival rates, and poorer prognosis (28, 29). However, Hashimoto et al. (30) found that T stage could not predict the risk of progression from biochemical recurrence to CRPC in patients with prostate cancer. This discrepancy may stem from differences in research methodologies and requires further validation through multicenter prospective clinical trials.

Peripheral blood immune markers, as non-invasive biomarkers, have demonstrated significant potential in early cancer screening, prognostic evaluation, and treatment response prediction for a variety of malignant tumors. The present study identified the PLR and SII as independent predictors of CRPC progression after endocrine therapy. The predictive value of these indices likely stems from their ability to comprehensively assess systemic inflammation and immune suppression. As novel peripheral blood-based inflammatory biomarkers, both PLR and SII have demonstrated significant clinical utility in predicting prostate cancer progression and prognosis. An elevated PLR reflects an imbalance between platelet activation and lymphocytopenia. Activated platelets enhance immunosuppression with the tumor microenvironment through pro-inflammatory factors, whereas a reduced lymphocyte count impairs immune surveillance. Together, these mechanisms drive CRPC progression. Relevant research has also confirmed that the PLR demonstrates clear diagnostic value in differentiating benign prostatic hyperplasia from prostate cancer. Moreover, in patients with metastatic CRPC, abnormal PLR elevation during abiraterone treatment was found to be independently associated with poor prognosis (hazard ratio =1.52, 95% CI: 1.16–1.98, P<0.001), indicating that dynamic PLR changes may further serve as a potential biomarker for assessing treatment sensitivity (31, 32). The SII comprehensively reflects the host immune–inflammation balance, with higher values indicating impaired immune function and increased tumor aggressiveness in cancer patients (33, 34). Relevant research has shown that a chronic inflammatory state in an organism can induce tumor initiation and progression, with a bidirectional relationship between the two (35). As a core parameter in multiparametric predictive models, the SII has demonstrated prognostic value in prostate cancer previously (36). The results of the present study further indicate that dynamic changes in the SII correlate with the risk of progression to CRPC within 2 years of ADT in prostate cancer patients, providing an objective basis for the early identification of high-risk patients.

During malignant tumor progression, the host immune system exerts antitumor effects through immune surveillance mediated by T lymphocytes and their subsets. The quantitative distribution characteristics of peripheral lymphocyte subsets can serve as dynamic monitoring indicators for evaluating the host’s immune–inflammatory status. Dysregulated distribution of lymphocyte subsets often reflects an imbalance in immune homeostasis, and this dysregulated state is significantly positively correlated with tumor immune evasion and clinical progression (37). CD4^+^ T cells generally augment antitumor immunity, whereas CD8^+^ T cells can exert inhibitory effects on the immune response (38). Under physiological immune homeostasis, the CD4^+^/CD8^+^ T cell ratio is maintained in a dynamic equilibrium by a finely tuned molecular regulatory network. The present study identified a decreased CD4^+^/CD8^+^ T cell ratio as an independent predictor of progression to CRPC within 2 years of endocrine therapy in prostate cancer patients. The underlying mechanism is likely linked to disrupted immune homeostasis and ADT-induced suppression of T-cell function. Although the absolute counts of CD3^+^, CD4^+^, and CD8^+^ T cells as well as B cells and natural killer cells showed no predictive value, the integrated CD4^+^/CD8^+^ T cell ratio more sensitively captures the relationship between tumor progression and its dynamic changes. This finding is consistent with the results reported by Smith et al. (39).

Our study demonstrates that an immune–inflammatory evaluation system based on routine peripheral blood indices, including the PLR, SII, and the CD4^+^/CD8^+^ T cell ratio, can effectively predict the progression of prostate cancer to CRPC, indicating that host systemic immune status represents a key, quantifiable dimension influencing disease outcomes, with unique translational value in clinical practice. Importantly, this immune–inflammatory system may serve as a valuable adjunct to molecular biomarkers recommended in current clinical guidelines (e.g., AR-V7, ctDNA, and HRR gene mutations). Molecular profiling enables precise characterization of tumor genomic drivers and informs long-term precision treatment strategies; however, its clinical application is often constrained by high costs, technical barriers, and limited feasibility for frequent dynamic monitoring (40). In contrast, the immune ratios evaluated in this study offer advantages of non-invasiveness, cost-effectiveness, and feasibility for high-frequency longitudinal monitoring. These two biomarker categories provide complementary information across distinct biological dimensions, with molecular biomarkers primarily defining “what the tumor is” (genotypic characteristics) and dynamic immune indices capturing in real time “how the host responds to the tumor” (immune phenotypic status). An integrative framework combining these two categories can offer a more comprehensive and precise system for CRPC risk stratification and longitudinal disease monitoring.

Nevertheless, it should be acknowledged that the PLR, SII, and the CD4^+^/CD8^+^ T cell ratio represent indirect surrogates of complex immune regulatory processes and do not directly represent the mechanisms through which immune dysregulation drives CRPC progression, constituting an inherent limitation of the present study. To enhance mechanistic insight and align with contemporary immuno-oncology paradigms, future studies should advance in two key directions. First, at the clinical application level, priority should be given to combined validation and integrative modeling of the immune indices identified in this study with molecular biomarkers, in order to quantify the incremental predictive value of their synergistic integration. Second, at the mechanistic level, it is essential to incorporate more direct assessments of immune function and the tumor microenvironment, including evaluation of T-cell functional states (e.g., exhaustion markers such as PD-1 and TIM-3, as well as activation-related markers), systematic profiling of circulating immunoregulatory cytokines (e.g., IL-6, TNF-α, and TGF-β), and longitudinal monitoring of immune indices across the treatment course. Such advanced immune profiling will be critical for delineating the specific contribution of immune dysregulation to the progression of advanced prostate cancer to CRPC. Ultimately, integration of multidimensional immune profiling, tumor genomic characteristics, and clinicopathological parameters is expected to enable the development of mechanism-driven precision prediction models, which will improve CRPC risk stratification and provide a robust theoretical foundation for novel combination immunotherapeutic strategies.

The present study has some limitations that should be considered when interpreting the results. This was a retrospective study with a relatively small sample size, which may have introduced selection bias and limited the statistical power. First, the model was developed based on retrospective data. Although internal validation yielded satisfactory results, independent external validation is still required to confirm its generalizability. Therefore, future efforts should focus on conducting multi-center external validation and further promoting prospective studies to evaluate its clinical translational value. Second, this study exclusively utilized baseline immune-inflammatory markers. As these markers (e.g., PLR, SII) are dynamic and can fluctuate due to therapy, host response, and changing conditions, our static, pre-treatment model may not capture critical predictive information related to treatment response or disease evolution, potentially limiting its temporal accuracy. Future research should track dynamic changes in immune status and PSA levels during treatment (e.g., fluctuations in lymphocyte subsets after ADT, PSA nadir, and time to PSA progression) to avoid missing critical predictive information.

In summary, this study developed a predictive model for progression to CRPC after endocrine therapy in patients with prostate cancer by integrating peripheral blood immune markers with tumor TNM staging. The developed model exhibited favorable predictive performance and clinical utility. Compared with traditional models incorporating only TNM staging and tumor markers, the inclusion of indicators reflecting systemic inflammation and preoperative immune status likely improved the prognostic prediction accuracy. The model performed well in internal validation, but external validation is still needed.

## Data Availability

The raw data supporting the conclusions of this article will be made available by the authors, without undue reservation.
